# A case report: retigabine induced oral mucosal dyspigmentation of the hard palate

**DOI:** 10.1186/s12903-015-0102-y

**Published:** 2015-10-09

**Authors:** Nicholas G. Beacher, Martin J. Brodie, Christine Goodall

**Affiliations:** Special Care Dentistry, University of Glasgow Dental School, School of Medicine, College of Medical, Veterinary & Life Sciences, 378, Sauchiehall Street, Glasgow, G2 3JZ UK; Medicine and Clinical Pharmacology, Western Infirmary, Epilepsy Unit, Western Infirmary, Glasgow, UK; Oral Surgery and Sedation. University of Glasgow Dental School, School of Medicine, College of Medical, Veterinary & Life Sciences, Glasgow, UK

**Keywords:** Retigabine, Oral mucosal dyspigmentation

## Abstract

**Background:**

Dyspigmentation of the oral mucosa has a multitude of aetiological causes. Retigabine, a new antiepileptic drug, has the potential side effect of inducing a blue/purple pigmentation of the oral mucosa in addition to the skin, lips, nails and retina of the eyes. This article presents a unique case of dyspigmentation present in the oral mucosa of the hard palate which has previously been unreported in the dental literature.

**Case presentation:**

A 70 year old white male presented to a secondary care oral surgery department with an unusual asymptomatic pigmented lesion present in the hard palate of the oral cavity. The pigmentation was remarkable for its distinct blue/purple colouration which was associated with a similar discolouration of the nail beds of the hands. This is believed to be a side effect of the anti-epileptic medication retigabine.

**Conclusion:**

The dental profession and wider healthcare community should be made fully aware of the potential side effect of oral dyspigmentation associated with the novel anti-epileptic medication retigabine. Enhanced knowledge of the causative role of retigabine in dyspigementation of the oral mucosa will allow the practitioner to make an appropriate diagnosis. As far the authors are aware this is reaction is unreported in the dental literature and should be disseminated to the wider oral health professional’s community.

## Background

Abnormal pigmentation of the oral mucosa can be disconcerting to the presenting patient and examining dental practitioner. The unexpected appearance of atypical mucosal tissue challenges the dentist to formulate a practical diagnosis, provide an explanation for the alerted mucosal form to the patient and where appropriate facilitate management. Mucosal dyspigmentation has a diverse aetiology varying from the nuances of physiological response through to pathological entities including malignancy.

Retigabine was licensed in Europe for use as adjunctive treatment for refractory partial epilepsy on 28^th^ March 2011. Drug resistant epilepsy is currently defined as the failure of adequate trials of 2 tolerated and appropriately chosen and tried antiepileptic drug (AED) schedules (whether as monotherapies or in combination) in order to achieve sustained freedom from seizures [1]. Despite acknowledged limitations in the epidemiological data [2] epilepsy is regarded as the most common global disorder of the brain with a reported prevalence of 7.3 in every 1000 people in the United Kingdom [3]. It is estimated that one third of those diagnosed with epilepsy fail to achieve seizure control and thus fall into the refractory epilepsy category [4]. This in turn has a significant negative impact upon quality of life [5].

Retigabine has shown much promise in the management of patients with refractory partial onset seizures [6, 7]. Medical intervention by its very nature challenges the prescribing clinician to juxtapose the benefit to a patient’s condition against the potential detrimental side effects of therapy. As with all medications, retigabine carries special warnings and precautions associated with its use. These include risk of malaise, nausea, renal and urinary disorders, dyspepsia and of particular interest to the dental practitioner peripheral oedema and dry mouth [8]. Occlular disturbance including blurred vision in addition to dizziness and tremors have also previously been identified [9]. Discolouration of the retina with or without similar discolouration of the nails, lips and skin have been reported and some concern exists that this effect may become permanent [10]. At present, few reports exist in the available literature which identify dyspigmentation of the oral mucosa as a potential consequence of the drug retigabine [11, 12].

We present a case of altered pigmentation of the oral mucsoa present on the hard palate in a 70 year old gentleman receiving treatment with retigabine for more than 2 years.

## Case presentation

A 70 year old male presented to The Oral Surgery Department at The Glasgow Dental Hospital and School in November 2014, three weeks following an urgent referral from his general dental practitioner.

The gentleman reported having an asymptomatic purple/blue lesion present on the roof his mouth. This was an incidental finding at his most recent 3 monthly regular dental appointment, not having been recognised prior to this.

The medical history was remarkable in that the patient had suffered from partial onset seizures from the age of 17 years as a consequence of right sided mesial temporal sclerosis. At the time of referral the patient was prescribed a drug regime of, retigabine 300 mg three times daily, primidone 250 mg in the morning and 125 mg in the evening, zonisamide 200 mg in the morning and 300 mg in the evening and clobazam 20 mg at night, to manage his partial onset seizure. The gentleman had initiated retigabine therapy in April 2012 and it had substantially improved his seizure control. Despite reporting falling asleep easily, he otherwise functioned well and thus was maintained on this drug regieme. Additionally, the patient had a diagnosis of osteoporosis, for which he was receiving alendronic acid and calcichew. He was also prescribed omeprazole in order to manage the dyspepsia associated with a hiatus hernia. He has never smoked or used tobacco products and consumes 10 units of alcohol per week. He regularly attends the dentist and does not wear an intra-oral prosthesis.

**Fig. 1:**
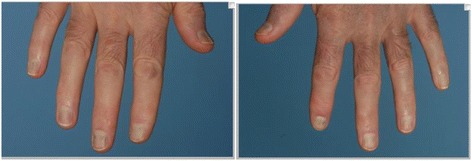
Purple/blue discolouration of the *left* and *right* nail beds of the hands

Extra-oral examination revealed a purple/blue discolouration of the nail beds of both the left and right hands (Fig. 1) and the lips were tinged with a bluish hue extending to the vermillion border. No alteration in the pigmentation of the retina of the eyes was observed on examination. No other skin abnormalities were reported by the patient or observed by the examining clinician.

**Fig. 2:**
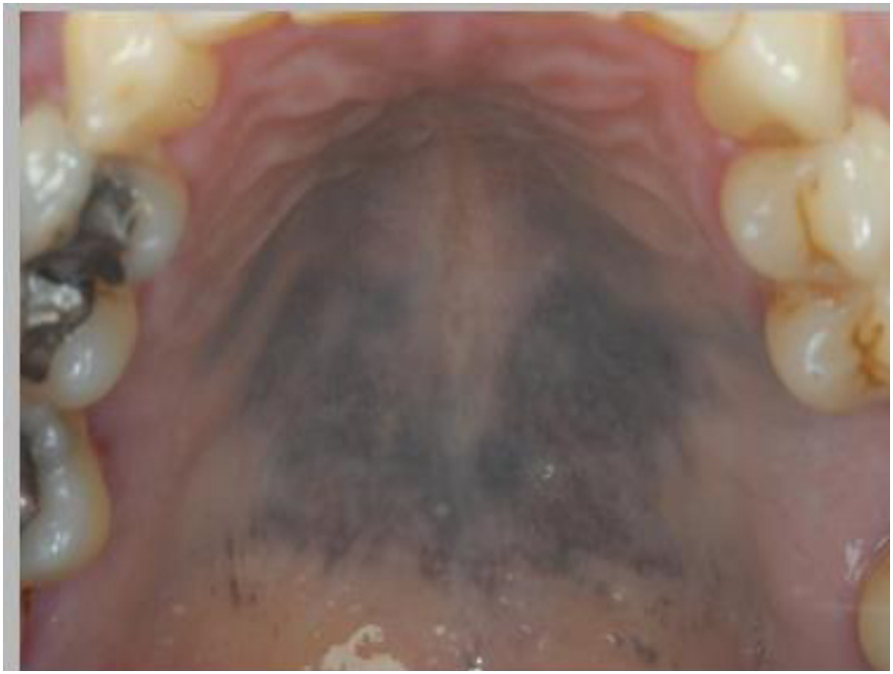
Purple/blue dyspigmentation of the mucosa of the hard palate

Intra-oral soft tissue examination revealed the mucosa of the hard palate was dyspigmented being a purple/ blue colour (Fig. 2). The colour observed was identical to that noted in the nail beds of the hands. The discolouration of the palate observed was uniform, extending symmetrically across the mucosa from the median palatine suture to the gingival margins on the palatal aspect of the teeth. The epithelium was intact and showed no other signs of abnormality other than the altered colour.

On further detailed questioning the gentleman reported that he had been aware of the discolouration of the nail beds but as they had caused him no symptoms he was unconcerned. The patient was able to recall that this altered colouration had developed within the previous 3 months. The timing of the appearance of the pigment within the nail beds is consistent with the development of the blue pigmentation within the oral mucosa, as the patient had received an oral examination 3 months ago where no changes in the oral mucosa were reported by the oral health professional.

No further special investigations were undertaken at this stage as the appearance of the mucosa observed was consistent with the skin pigmentation observed and corresponded with available reports. Temporal factors were important to the formulation of the diagnosis as the pigmentation observed only became evident subsequent to the patient beginning therapy with the drug retigabine. Furthermore, the accepted side effect of pigmentation of the nail beds appears to have occurred within the same time period as the discolouration of the oral mucosa. The notable similarity in colour of the dyspigmented skin and oral mucosa further supported the diagnosis.

Other possible diagnoses were excluded based on clinical expertise. A vascular cause was considered however, no blanching was observed when pressure was applied to the mucosa nor was there any increase in cellular number or size which would clinically present as an abnormal lump or growth.

Melanoma, typically presents as invasive or raised lesion with a dark-brown or bluish-black pigmentation. The lesion observed was notably distinct from this in both colouration and the intact nature of the mucosa.

Similarly, Kaposi’s sarcoma, a lesion synonymous with H.I.V., was ruled out as a possible diagnosis as typical features such as a reddish purple pigmentation and nodular surface were absent. Furthermore, the patient did not report having a blood borne virus nor did he display any signs of being in a high risk group for a blood borne virus.

It is the belief of the authors that blue pigmentation was an extension of the previously reported skin alteration. Biopsy was considered however when taking all factors into consideration surgical sampling was deemed not to be justified. The clinical features observed were considered to be consistent with the appearance of pigmented change as a consequence of retigabine’s use at other sites reported within the medical literature and together with the absence of confirmatory diagnostic histopathological criteria for retigabine induced dyspigmentaion a clinical decision not to biopsy the mucosa was made [11]. Other possible systemic pathological causes were excluded on clinical grounds and recent blood sampling showed no irregularities indicating the need for further investigation.

The patient was reviewed six weeks following initial examination. There were no notable changes in the patient’s symptoms or the clinical appearance of the mucosa. A further six month review is scheduled when the clinical appearance of the oral mucosa will be re-evaluated.

## Discussion

Dyspigmentation of the oral mucosa has a varied aetiology which must be fully considered by the diagnosing clinician. Oral manifestations of systemic disease can present as altered mucosal pigmentation including: Addison’s disease, Cushing’s syndrome, Peutz-Jeghers syndrome [13] and McCune Albright syndrome [14]. Addison’s disease, an endocrine pathology of the adrenal gland results in decreased cortisol and aldosterone production. Darkening of the skin, much like the appearance of a sun tan is one of the most notable features. Other symptoms include malaise, muscle weakness, unintentional weight loss and increased thirst [15]. Further investigation for diagnostic purposes would include measuring blood cortisol levels and if sufficiently low an ACTH simulation test may be indicated. The person with correctly diagnosed Addison’s disease would have no increase in the levels of hormone secreted following administration of the ACTH.

Cushing’s syndrome, another endocrine disease, results in an increased secretion of ACTH from the pituitary gland as a result of long term steroid use (iatrogenic) or due to disease of the gland itself (endogenous). Symptoms include weight gain with associated fatty deposits of the face resulting in characteristic “cushinoid features” and the appearance of red/purplish markings on the proximal aspects of the body. Special investigations attempt to measure the level of cortisol using a urine test, salivary sampling or a steroid suppression test [16].

However, variation in the pigmentation of the oral mucosa can be entirely benign, exemplified in the oral mucosa of those darker skinned racial groups as a result of entirely normal physiological melanin production. The variation in pigmentation is most pronounced in the anterior aspects of the mouth and tends to increase in both intensity and surface area with increasing age [17]. Other melanocytic lesions tend to present in a focal region of the mouth and include melanocytic macule, melanocytic naevus, melancanthoma, smoking induced melanosis and melanoma [18].

A discolouration of the oral mucosa can result from the deposition of exogenous materials. Common to the dental practitioner is the amalgam tattoo which presents as a localised grey region in the mucosa as a result of amalgam fragments being introduced into the tissues following tooth restoration or extraction [19]. Historically, other heavy metals including bismuth and lead resulted in a linear patterned blue/black pigmentation which follows the gingival contour as precipitates are exuded in gingival crevicular fluid as a consequence of occupational exposure or medical therapies [20].

A growing number of reports of oral post inflammatory pigmentation are appearing in the literature. Following chronic inflammatory states present in the oral tissues, such as that induced by lichenoid reactions and lichen planus, excess deposits of melanin develop in the epithelial basal layer and surrounding connective tissue of the oral mucosa [21].

A multitude of medications are cited as inducing colour change within the tissues of the mouth. The antimalarial drugs, oral contraceptives and cytotoxic agents all upregulate melanin production resulting in oral pigmentation [13]. The tetracyclines, in particular minocycline, may also induce a similar change. However, this is due to staining of the underlying hard tissues not as a consequence of increased melanin present in the mucosa [22].

The medication retigabine, a therapy used for the management of drug-resistant epilepsy, is the first available neuronal potassium channel opening drug [23]. It acts to improve the stability of neurones, subsequently preventing seizures [24]. The results obtained with retigabine in phase III regulatory trials have been positive in the clinical management of refractory epilepsy [6, 7]. It is unfortunate that the significant side effects associated with retigabine have resulted in the European Medicines Agency (EMA), to recommend retigabine as a last resort therapy [25]. This recommendation is primarily as a result of the pigmentation of the skin, nails, lips and eye tissues which can develop following its use.

The findings in this case report provide further evidence that retigabine induces a blue purple pigment change to the skin and mucosa including the oromucosal tissue of the hard palate. There is only one previous publication, which the authors are aware of that has noted changes in the oral mucosal tissue, [11]. It is important that the dental profession is aware of this new drug reaction in order to provide a diagnosis and be able to inform patients.

The mechanism in which this pigmentation is induced is as yet unknown. Recent studies in rats have implicated pigmented dimerization products of retigabine in producing the discolouration [26]. The histopathological features reported in a biopsy sample of dyspigemtend mucosa of the hard palate by Shkolnik, showed normal epithelium without any melanin pigmentation [11]. However, melanin was evident in the submucosal macrophages and extracellular matrix. Further testing to isolate the drug in the mucosal sample using nuclear magnetic resonance and mass spectrometry were unable to identify the presence of retigabine directly within the specimen. The group propose a “Tyndall effect” to explain the appearance of the pigmented mucosa. The blue clinical appearance of melanin is explained as a result of short wavelength blue light being scattered the most when compared to other wavelengths.

No active treatment is required to manage this oral pigmentation. However, liason with medical colleagues should facilitate an opportunity for the physician and patient to re-evaluate continuation of therapy to manage partial-onset seizures.

Patients who exhibit extra-oral features of this reaction should be encouraged to avoid and protect the skin from sunlight as this may well precipitate the reaction. It is unclear at present if the pigmentation will resolve following cessation of the medication. Shkolnik’s reported case did show improvement in the pigmentation following discontinuation of retigabine. Although, the F.D.A. report suggests that the pigmentation has the potential to be permanent [10], whilst Scholnik's report suggested that the problem may reversible on stopping the drug [11]. In this presented case it is possible but unlikely that the other medications taken by the patient have a role in initiating or perpetuating the dyspigmentation present in the oral mucosa. It is clear further research is required to increase the understanding of such reactions and their long term outcomes.

## Conclusions

The clinical presentation of abnormal oral mucosal pigmentation is a challenge for the examining clinician to accurately diagnose. The dental practitioner should have a thorough understanding of the causes of oral mucosal lesions and causes of pigmentation. They must be able to facilitate diagnosis and instigate referral to specialist services where necessary. The AED, retigabine is a new medication which the practicing dental professional should be aware of as it can result in dyspigmentation of the mucosa of the hard palate in addition to the nails, skin and retina of the eyes.

## Consent

Written informed consent was obtained from the patient for publication of this Case report and any accompanying images. A copy of the written consent is available for review by the Editor of this journal.
